# The first report of *Haplomitriumgibbsiae* (Steph.) R.M. Schust. (Haplomitriaceae) from Guangxi, China

**DOI:** 10.3897/BDJ.13.e155213

**Published:** 2025-06-12

**Authors:** Wei Han, Haifeng Luo, Yuqing Li, Nanqiang Li, Fei Tan, Xiangju Huang, Qiang He

**Affiliations:** 1 School of Resources and Chemical Engineering, Sanming University, Sanming, China School of Resources and Chemical Engineering, Sanming University Sanming China; 2 College of Chemistry and Materials Science, Fujian Normal University, Fuzhou, China College of Chemistry and Materials Science, Fujian Normal University Fuzhou China; 3 Jiuwanshan National Nature Reserve Administration of Guangxi, Liuzhou, China Jiuwanshan National Nature Reserve Administration of Guangxi Liuzhou China; 4 State Key Laboratory of Systematic and Evolutionary Botany, Institute of Botany, Chinese Academy of Sciences, Beijing, China State Key Laboratory of Systematic and Evolutionary Botany, Institute of Botany, Chinese Academy of Sciences Beijing China

**Keywords:** liverworts, *Haplomitriumgibbsiae*, China, new record, biogeography

## Abstract

**Background:**

The liverwort genus *Haplomitrium*, first described by Nees in 1833, is the basal sister group to all other liverworts. It exhibits distinctive traits, including upright shoots, radial leaf arrangement, abundant mucilage and the absence of rhizoids, reflecting its non-vascular nature. Predominantly found in the Southern Hemisphere, specifically Australasia, the genus comprises seven species and two infraspecific taxon globally, with China recording three species — *H.blumei*, *H.mnioides* and *H.hookeri*. These features and its distribution underscore its significance in studying early terrestrial flora.

**New information:**

The liverwort species *Haplomitriumgibbsiae* (Steph.) R.M. Schust., previously considered endemic to Gondwanan regions, has been documented in East Asia for the first time during a floristic survey in the Jiuwanshan National Nature Reserve, Guangxi Zhuang Autonomous Region, China. This discovery expands the known range of the species and, when combined with prior hypotheses, suggests a potential dispersal pathway: following the fragmentation of Gondwana, *H.gibbsiae* may have spread to India and South America and subsequently from India to East Asia via the Himalayas, resulting in its present-day global distribution. Additionally, based on extensive literature review, the present study discusses the species’ retention of primitive traits and considers its potential as a candidate for future research, aimed at deepening our understanding of early land plant evolution.

## Introduction

The liverwort genus *Haplomitrium* (Haplomitriaceae, Calobryales), was firstly described by Nees (1833). As a basal sister group to all other liverworts, the genus holds a pivotal position in the classification and evolutionary study of the lineage (Qiu et al. 2006), shedding light on the morphological and ecological adaptations of early land plants (Renzaglia et al. 2000). Species of *Haplomitrium* are characterised by a suite of distinctive features that confirm its classification as a non-vascular plant and provide insights into the evolutionary history of early terrestrial flora (Campbell 1895). These features include a branched, rhizoid-less rhizome giving rise to slender, upright shoots with a three-ranked leaf arrangement, abundant mucilage along stems and leaf margins, conspicuous sexual organs atop the erect stems and the absence of perianths, which also distinguish *Haplomitrium* from other liverwort genera (Bartholomew-Began 1991). The genus exhibits a broad distribution across temperate, subtropical and tropical regions, spanning North America, the West Indies, South America, Europe, Asia, Africa, Australia and the Pacific Islands (BFNA 2025), with its greatest species diversity concentrated in the Southern Hemisphere, particularly Australasia (Bartholomew-Began 1991). Although Engel and Glenny (2008) proposed recognising 15 species within the genus, most taxonomic revisions have refined this to a widely accepted total of seven species and two infraspecific taxon worldwide (Bartholomew-Began 1991, Forrest et al. 2006, Crandall-Stotler et al. 2009, Söderström et al. 2016, Stotler and Crandall-Stotler 2017, WFO 2025).

In China, botanical records have identified three species of *Haplomitrium*, reflecting a modest, but significant diversity within the genus. These include *Haplomitriumblumei* (Nees) R.M. Schust., recorded in various studies (e.g. Yang (1966), Gao and Li (1985), Gao (2003), Gao and Wu (2010), Zhang et al. (2011), Jia and He (2013), Sheng et al. (2022)); *Haplomitriummnioides* (Lindb.) R.M. Schust., extensively reported across multiple provinces of the country (e.g. Yang (1966), Gao and Li (1985), Gao and Cao (2000), Shi and Zhu (2006), Gao and Wu (2010), He et al. (2010), Zhang et al. (2011), Jia and He (2013), Wu and Zhang (2013), Cheng and Shi (2019), Yu et al. (2019)); and *Haplomitriumhookeri* (Lyell ex Sm.) Nees, documented in fewer instances (Higuchi et al. 2000, Jia and He 2013). The records of *Haplomitrium* in China, spanning from the mid-20^th^ century to recent years, demonstrate a sustained interest in the taxonomy of this genus. However, the uneven frequency of citations may indicate potential gaps in regional surveys or ecological representation, which merit further investigation.

During a recent floristic survey in the Jiuwanshan National Nature Reserve, Guangxi Zhuang Autonomous Region, China, we discovered *Haplomitriumgibbsiae* (Steph.) R.M. Schust., a species originally described from New Zealand by Stephani (1917) and previously known only from Gondwana-derived regions (Bartholomew-Began 1991). This finding marks the first record of *H.gibbsiae* in East Asia which formed by the breakup of Laurasia, significantly expanding its known distribution.

## Materials and methods

A specimen of *Haplomitriumgibbsiae* (Steph.) R.M. Schust. was collected on 28 May 2024, with collection number 16957, by Q. He from the Institute of Botany, Chinese Academy of Sciences, Beijing, in the Yangmei’ao of the Jiuwanshan National Nature Reserve in Yongle Town, Rongshui Miao Autonomous County, Liuzhou City, Guangxi Zhuang Autonomous Region, China, at coordinates 25.20349°N, 108.67904°E with an altitude of 1200 m. The specimen was identified by Q. He and subsequently deposited in the Herbarium (PE) at the Institute of Botany, Chinese Academy of Sciences, Beijing, under catalogue number PE80000632.

We conducted morphological observations on the specimen using light microscopy. Photographs of the plant's external morphology were captured with a stereoscopic zoom microscope SMZ1000 with the eyepieces C-W10xB/22 (Nikon Corporation, Tokyo, Japan) coupled with a Nikon C-0.55x DS C-Mount adapter. Microscopic pictures were acquired and measured with a Leica DM4000 B LED microscope along with a digital microscope camera Leica DFC450 (Leica Microsystems, Wetzlar, Germany). All digital images were processed and arranged with Adobe Photoshop Elements 10 software (Adobe Systems Incorporated, California, USA).

To construct a current global distribution map of *H.gibbsiae*, we compiled occurrence data from multiple sources, integrating the newly-reported East Asian distribution from this study with previously documented ranges in Australia, New Zealand, Chile and India. We obtained one record with geographic coordinates for India from the Tropicos (2025). Additionally, we retrieved 91 occurrence records for Australia, New Zealand and Chile from the GBIF (2025), all of which contained precise geographic coordinates. Ultimately, we retained 93 records with valid geographic coordinates, comprising seven for Australia, 72 for New Zealand, 12 for Chile, one for India and one for China. We used QGIS version 3.40.4-Bratislava (QGIS Development Team 2024) to generate the distribution map.

## Taxon treatments

### Haplomitrium gibbsiae

(Steph.) R.M. Schust, 1917

80D51B57-AB00-550F-97EE-39A51D86B7C4

#### Materials

- **Type status:** Other material. **Occurrence:** catalogNumber: PE80000632; recordNumber: 16957; recordedBy: Q. He; occurrenceID: 7816BD46-02B2-5258-A25E-8CBD0EF2BDB5; **Taxon:** class: Haplomitriopsida; order: Calobryales; family: Haplomitriaceae; **Location:** continent: Asia; country: China; countryCode: China/CN; stateProvince: Guangxi Zhuang Autonomous Region; county: Rongshui Miao Autonomous County; municipality: Yongle Town; locality: Yangmei’ao; verbatimLocality: By the roadside; verbatimElevation: 1200 m; verbatimLatitude: 25.20349°N; verbatimLongitude: 108.67904°E; **Identification:** identifiedBy: Q. He; **Event:** year: 2024; month: 5; day: 28; habitat: Growing along the roadside by the edge of the forest, rooted in thin soil and clinging to the rocky cliffs.; **Record Level:** language: cn

#### Description

**Figure 1.**
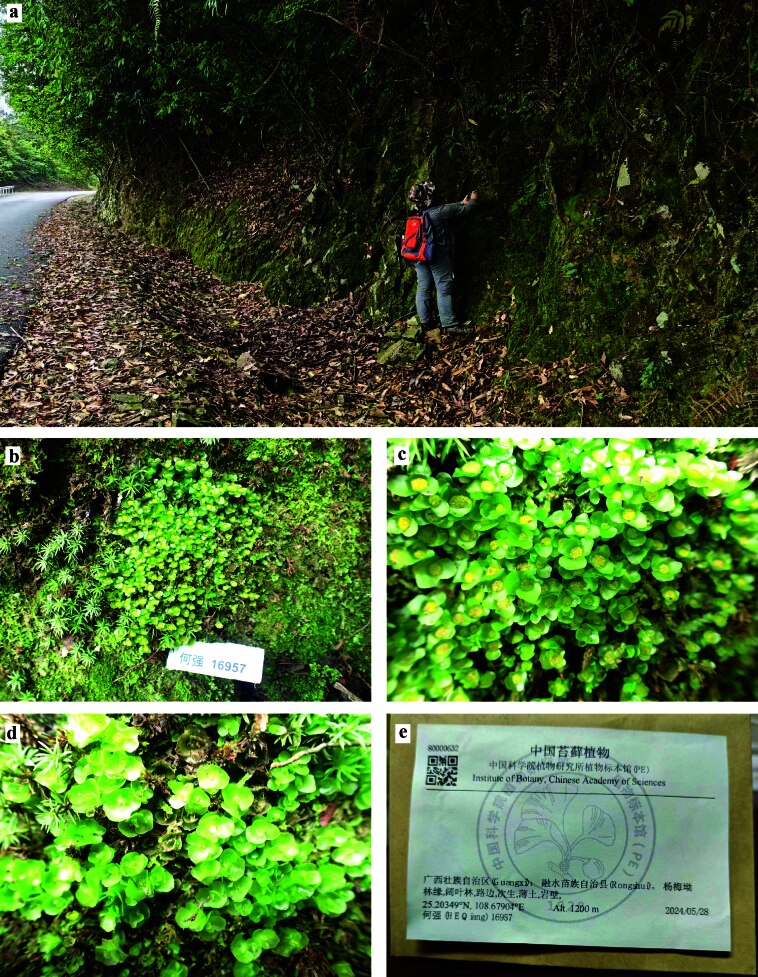
*Haplomitriumgibbsiae* (Steph.) R.M. Schust. in the wild. **a** Habitat; **b-d** Population; **e** Voucher specimen. All from *HE Qiang 16957*.

**Figure 2.**
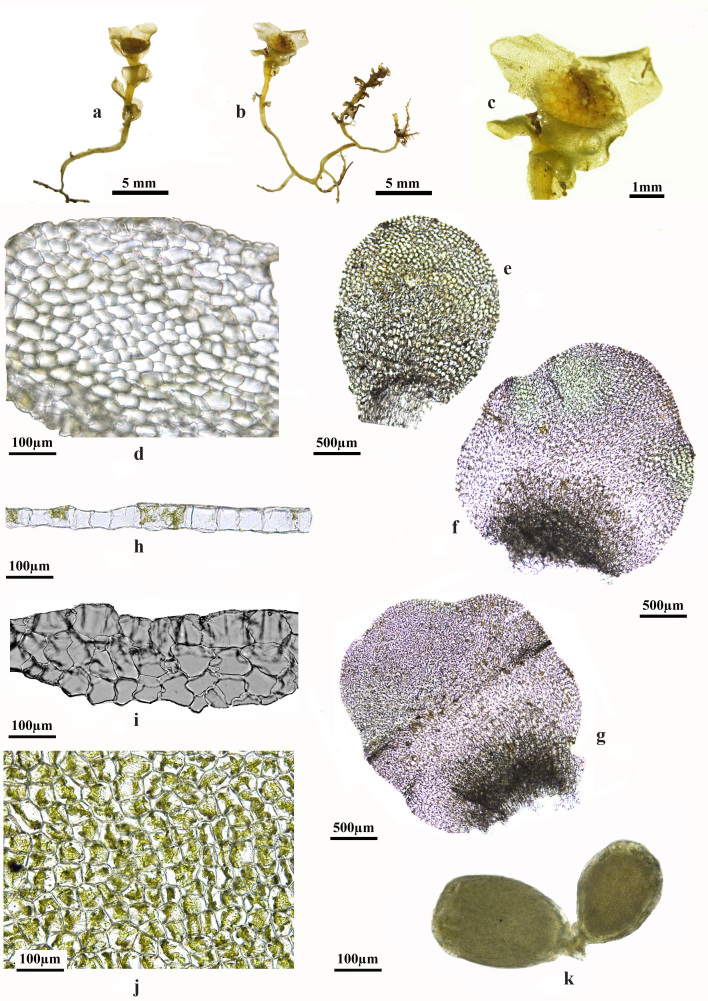
*Haplomitriumgibbsiae* (Steph.) R.M. Schust. **a, b** Plant with leaves; **c** Antheridia with bracts; **d** Transverse section of stem; **e-g** Leaves; **h** Transverse section of leaf (mid-region); **i** Transverse section of leaf (base); **j** Cell with oil body of leaves; **k** Antheridium. All from *HE Qiang 16957*.

The species exhibit variable morphology, with stolons arising from a prostrate, irregularly branched (Fig. 1b-d), colourless to ivory-coloured rhizome that often appears coralloid (Fig. 2b) and is adorned with numerous 1-2-celled slime papillae secreting a thick mucilaginous sheath. Erect leafy shoots arise from this rhizomatous system, displaying isophyllous or rarely anisophyllous arrangement with infrequent terminal branching (Fig. 2a-b). The leaves, bright yellow-green, translucent to opaque, rigid and brittle, are arranged in three subequal to equal ranks; lateral leaves are weakly succubously to nearly transversely inserted, often strongly undulate or crispate and widely spreading (Fig. 2e-i). Leaf shape varies from suborbicular-ovate basally to deltoid, rhomboidal or rhomboidal-reniform distally, typically bluntly pointed or acute at the tips, widest in the basal quarter and broader than long, with bluntly pointed or acute tips and undulate to crispate margins. Unistratose, except for a 2-4 stratose basal field, they have entire margins, occasionally bearing vestigial teeth or slime papillae near reproductive zones that fade with age. Median leaf cells range from 40-65 μm wide by 45-85 μm long (Fig. 2h-i). Leafy stems, unbranched above and nearly colourless and leafless below, with scattered, remote leaves that enlarge abruptly distally (Fig. 2a-b). Oil bodies, hyaline and finely granular, occupy a small cell portion (Fig. 2j).

The dioecious plant bears gametangia typically aggregated in distinct terminal rosettes, rarely scattered along the stem, with apical proliferation variably present (Fig. 1b-d and Fig. 2b). Male plants produce numerous antheridia (25-45 or more per androecium) in terminal receptacles, technically axillary within the cycle of male bracts, which are initially ivory-white, transitioning to cinnamon with age (Fig. 2c and k). Female plants produce approximately 20 archegonia aggregated in a terminal receptacle (Engel and Glenny 2008). The perichaetium varies from rhombic to subrotund or irregularly rhomboid-ovate, often sinuous-crispate, initially erect and convolute, becoming strongly spreading at maturity (Engel and Glenny 2008). The sporophyte, protected by a perigynium bearing archegonia, bractlets and slime papillae (bractlets absent or raised to the basal one-fifth to one-third), is usually solitary, though 2-4 may develop per gametophore; the seta extends 20-25 mm above the perigynium, supporting a capsule (0.5 × 3-4 mm) that dehisces along (2-3-)4(-5) incomplete lines (Bartholomew-Began 1991). Spores, 20-39 μm in diameter, often remain in tetrads or diads until late development, with a coarsely verrucate exine (verrucae 1.7-3.2 μm high), pale brown and adorned with irregular, roughened or tuberculate compound markings. Elaters, 7-10 μm wide × 280-520 μm long, are predominantly 2-spiralled (occasionally 3-spiralled), transitioning to 1-spiralled at the tips or throughout (Bartholomew-Began 1991, Engel and Glenny 2008).

#### Distribution

**Figure 3.**
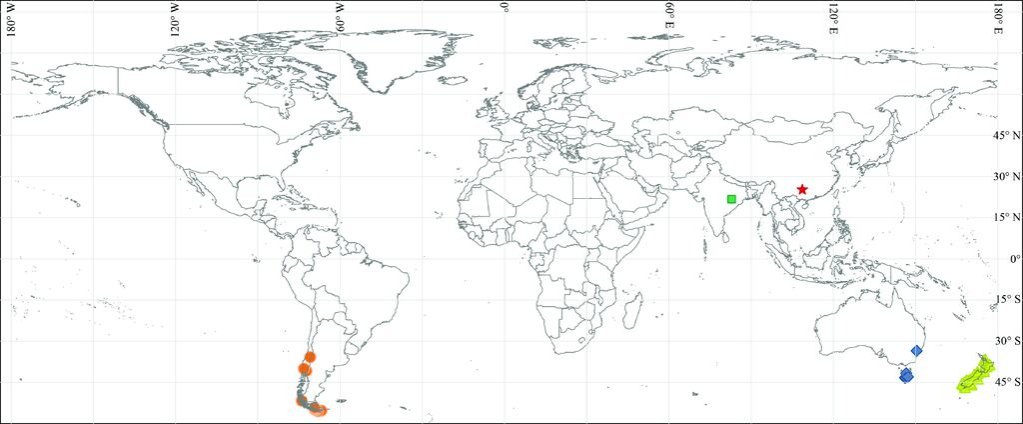
The worldwide distribution map of *Haplomitriumgibbsiae* (Steph.) R.M. Schust. (yellow triangles, blue diamonds, orange dots, green square and red star, represent localities of New Zealand, Australia, Chile, India and China, respectively).

The newly-documented occurrence of *H.gibbsiae* from China, reported in this study, which, when integrated with previously established records from New Zealand (Gibbs 1911, Campbell 1959, Schuster 1967, Allison and Child 1975), Australia (Engel 1981), India (Udar and Chandra 1961, Kumar and Udar 1976, Kumar and Udar 1977, Udar and Kumar 1982), Chile and Argentina (Hässel de Menéndez 1970, Schuster 1971, Hässel de Menéndez and Solari 1975), sourced from the Tropicos and GBIF, yield a total of 93 records with valid geographic coordinates for the species. Amongst these, New Zealand accounts for the highest number of distribution records, totalling 72, followed by Chile and Australia, with 12 and seven records, respectively. This comprehensive dataset supports the development of an updated global distribution map (Fig. 3), which illustrates the expanded range of *H.gibbsiae* to include East Asia, alongside its previously documented presence in the Southern Hemisphere and the Indian subcontinent.

#### Ecology

In New Zealand, *H.gibbsiae* occupies a diverse array of habitats, ranging from moist, disturbed sites such as clay banks and swampy *Sphagnum* areas to alpine environments where it grows on bare, wet soil amongst small rocks, spanning elevations from sea level to at least 1280 m (Gibbs 1911, Campbell 1959, Schuster 1967). Engel and Glenny (2008) further noted that populations in New Zealand are predominantly associated with fresh mineral soil on soil banks formed by human activities — such as road construction, track development, slips or washouts — occurring in both open sites and forested settings, particularly in areas that have experienced anthropogenic disturbance. In southern South America, the species is typically found at sea level, often in turfs on soil within shady, damp woods (Hässel de Menéndez 1970, Schuster 1971, Hässel de Menéndez and Solari 1975). In India, *H.gibbsiae* is confined to the Darjeeling Region of the Eastern Himalayas, where it grows on shaded, sandy soil over rocks at elevations between 1969 and 2121 m (Udar and Chandra 1965, Kumar and Udar 1976, Kumar and Udar 1977, Udar and Kumar 1982). The newly-discovered Chinese population of the species was found at an elevation of 1200 m, growing on thin soil over rock walls at the edge of a broad-leaved forest (Fig. 1a, e). This habitat aligns closely with the species’ established ecological preferences, reflecting its consistent affinity for bare or thin soil conditions across both natural and disturbed environments as observed in its New Zealand distribution.

#### Taxon discussion

Initially described by Stephani (1917) as *Calobryumgibbsiae* St. from New Zealand, it was later reclassified by Schuster (1963) into the *Haplomitrium*. Subsequent revisions further clarified its status: Hässel de Menéndez and Solari (1975) synonymised the South American *Haplomitriumchilense* R.M. Schust. with the species and Bartholomew-Began (1991) subsumed several Indian taxa — *Haplomitriumdentatum* (D. Kumar & Udar) J.J. Engel, *Haplomitriumgrollei* D. Kumar & Udar, *Haplomitriumindicum* (Udar & V. Chandra) R.M. Schust. and *Haplomitriumkashyapii* Udar & D. Kumar — under the same species.

According to Bartholomew-Began (1991), *H.gibbsiae* exhibits similarities to *Haplomitriumintermedium* Berrie in both symmetry and habit. However, it is distinguished by its leaves, which feature extensive multistratose basal fields (Fig. 2i) — a characteristic that could set *H.gibbsiae* apart from all other *Haplomitrium* taxa (Bartholomew-Began 1991). Additionally, Schuster (1963) reported that the archegonia of *H.gibbsiae* are typically arranged in a terminal group, with the shoot capable of continued growth in the absence of fertilisation. In contrast, that of *H.intermedium* may be scattered along the axis, though they are usually grouped distally. Regrettably, the specimen collected in this study was male, preventing direct observation of archegonial characteristics.

Historically, *H.gibbsiae* was thought to be restricted to regions such as Australasia (Gibbs 1911, Campbell 1959, Schuster 1967, Allison and Child 1975, Engel 1981), South America (Hässel de Menéndez 1970, Schuster 1971, Hässel de Menéndez and Solari 1975) and India (Hässel de Menéndez 1970, Schuster 1971, Hässel de Menéndez and Solari 1975), all of which trace their geological origins to the breakup of Gondwanan (Schuster 1972, Stech and Frey 2004). Its unexpected discovery in China, a region of East Asia shaped by the fragmentation of Laurasia, significantly expands its known geographic range (Fig. 3).

#### Notes

Due to the limited phylogenetic studies on *Haplomitrium* available at their time, Engel and Glenny (2008) based their treatment of the genus on adaptations and modifications of earlier works by Schuster (1967) and Schuster (1971), rather than the study by Bartholomew-Began (1991). The latter employed experimental culture techniques, along with light and electron microscopy, to investigate the ontogenetic patterns underlying the morphological features of *Haplomitrium*, earning widespread recognition in subsequent taxonomy (Forrest et al. 2006, Crandall-Stotler et al. 2009, Söderström et al. 2016, WFO 2025). A key highlight of our work is the first documented record of *H.gibbsiae* in China, where a population was found thriving at the edge of a broad-leaved forest. Notably, the collected specimen here was male, with no female plants observed, leaving the morphological characteristics of female plants to be inferred from descriptions by Bartholomew-Began (1991) and Engel and Glenny (2008).

## Identification Keys

### Key to species of *Haplomitrium* in China

**Table:** 

1	Leaves with an extensive polystratose basal field	*H.gibbsiae*
–	Leaves with a small bistratose zone restricted to the extreme base	2
2	Leaves never divided completely to the base	*H.hookeri*
–	Leaves (at least some) divided almost completely to the base	3
3	Leafy shoots prostrate	*H.mnioides*
–	Leafy shoots erect	*H.blumii*

## Discussion

The present study reports the first occurrence of *Haplomitriumgibbsiae* (Steph.) R.M. Schust. in China, representing a significant extension of its known geographic range into East Asia. Previously documented only in New Zealand, Australia, southern South America and India, this finding challenges the long-held assumption of an exclusively Gondwanan distribution and points to a more complex biogeographic history that may encompass Laurasia-derived regions. Beyond its distributional novelty, *H.gibbsiae* offers critical insights into the evolutionary origins of land plants through its retention of primitive traits, including a unique reproductive strategy and endophytic fungal associations.

Extensive research by numerous bryologists has provided a comprehensive understanding of *H.gibbsiae*’s biology, spanning its cellular structure, reproductive processes, ecological interactions and taxonomic placement. Studies on its microscopic organisation (e.g. Campbell (1959), Grubb (1970), Carothers and Rushing (1988)) have elucidated its developmental intricacies, while investigations into its reproductive and morphogenetic features (e.g. Taylor et al. (1974), Bartholomew-Began (1991), Renzaglia et al. (2015)) have detailed its spore-producing mechanisms. Ecological and biochemical analyses (e.g. Markham (1977), Carafa et al. (2003)) have further revealed its environmental adaptations, complemented by taxonomic refinements (e.g. Stech and Frey (2004), Forrest et al. (2006)). These multidimensional efforts collectively underscore the species’ significance as a window into the evolutionary adaptations of early land plants.

Central to its evolutionary importance, *H.gibbsiae* exhibits several primitive traits that illuminate the ancient origins of embryophytes. Its reproductive strategy features the production of spores as permanent dyad pairs, a characteristic suggestive of non-simultaneous sporogenesis potentially linked to charophytic algae, the precursors of terrestrial plants (Renzaglia et al. 2015). Additionally, the species forms a distinctive endophytic symbiosis with aseptate fungi within its subterranean gametophytic axes, characterised by intracellular arbuscules and epidermal fungal swellings reminiscent of arbuscular mycorrhizal associations in higher plants (Carafa et al. 2003), which positions *H.gibbsiae* as a valuable candidate for exploring one of the earliest plant-fungal interactions. Structurally, its capsule walls display deep schizogenous fissures, providing clues to the evolutionary development of stomatal mechanisms, while its hydrophobic elaters, coated with lipid droplets, reflect an early adaptation for spore dispersal through capsule desiccation (Duckett and Pressel 2019). Together, these features establish *H.gibbsiae* as a pivotal species for understanding the primitive characteristics and evolutionary trajectory of land plants.

The disjunctive distribution of *H.gibbsiae* — now spanning Australasia, South America, India and East Asia — raises intriguing questions about its dispersal mechanisms. Although its hydrophobic elaters facilitate spore release (Duckett and Pressel 2019), the spores are short-lived, germinating within a month and unable to endure prolonged desiccation (Bartholomew-Began 1991). Their fragile, thin exine layer further renders them ill-suited for long-distance dispersal or persistence as perennating structures (Renzaglia et al. 2015). These biological constraints suggest that spore dispersal alone cannot account for the species’ widespread occurrence, prompting consideration of historical biogeographic processes. Schuster (1972) proposed that *Haplomitrium* originated in Gondwana, with Australasia as its primary centre of diversity, where six of the seven known species, including three endemics, are found (Bartholomew-Began 1991). *H.gibbsiae*, exhibiting reduced polymorphism, occupies peripheral regions such as South America and India. Schuster (1972) hypothesised that, during the Mesozoic, as the Indian Plate migrated and collided with the Eurasian Plate to form the Himalayas, *H.gibbsiae* reached the Indian subcontinent, subsequently spreading into the Eurasian landmass. Over millions of years, selective extinctions, likely driven by changing climatic conditions, facilitated its dispersal into East Asia, resulting in its current global distribution pattern, as evidenced by the present study, while comprehensive molecular analyses are needed to explore these dispersal mechanisms. Moreover, during data compilation, two records from GBIF (2025) and NYBG (2025), catalogued as 04434930 and 04434929, were identified with location remarks indicating "Northern Asia". Although lacking detailed provenance, these records provide tantalising evidence of a possible historical presence in Laurasia, further enriching the species’ biogeographic narrative.

Comparatively, *Haplomitrium* and *Takakia* S. Hatt. & Inoue stand out as some of the earliest diverging lineages within liverworts and mosses, respectively, sharing morphological traits, such as rhizomatous stems without rhizoids, tetrahedral apical cells producing three-ranked leaves and specialised water-conducting cells (Renzaglia et al. 2018). Cladistic analyses suggest a deep evolutionary connection between the two genera (e.g. Schuster (1984), Ligrone et al. (2000), Shaw and Renzaglia (2004)). Recent integrative research on *Takakialepidozioides* S. Hatt. & Inoue by Hu et al. (2023) has advanced our understanding of its adaptive evolution in the Tibetan Plateau, yet *Haplomitrium*, such as *H.gibbsiae*, remains underexplored despite its parallel significance. With its striking similarities to *Takakia*, *H.gibbsiae* emerges as a promising candidate for future studies to unlock critical insights into the early evolution and biogeography of land plants.

In conclusion, the discovery of *H.gibbsiae* in East Asia marks a novel expansion of its range into a Laurasia-derived region, offering fresh insights into its biogeographic history. This finding corroborates previous hypotheses that the species likely colonised India and South America following the breakup of Gondwana and subsequently dispersed to East Asia via the Himalayas, thus establishing its current global distribution. Beyond its biogeographic implications, the primitive traits of *H.gibbsiae* provide profound clues to the evolutionary origins of land plants, while its parallels with *Takakia* underscore its comparable significance amongst early diverging lineages. As such, *H.gibbsiae* holds immense potential as a notable point for future research, particularly through molecular approaches, to deepen our understanding of the evolution and dispersal of early land plants across the globe.

## Supplementary Material

XML Treatment for Haplomitrium gibbsiae
